# Primitive Neuroectodermal Tumor with Glioblastoma Multiforme Components in an Adult: A Collision Tumor

**DOI:** 10.7759/cureus.456

**Published:** 2016-01-11

**Authors:** Victoria Forbes, James Vredenburgh

**Affiliations:** 1 Medicine, University of Connecticut Health Center; 2 Hematology and Oncology, Saint Francis Regional Cancer Center

**Keywords:** primitive neuroectodermal tumor, glioblastoma multiforme, collision tumor, adult, temozolomide, craniospinal radiation, platinum-based chemotherapy

## Abstract

We report a rare case of a central nervous system collision tumor in a 40-year-old woman. Histopathological examination of her large temporal tumor revealed two different components making up the tumor tissue. The predominant component of the tumor was found to be a primitive neuroectodermal tumor. The other component was glioblastoma multiforme. Both of these tumors carry a poor prognosis, and primitive neuroectodermal tumors are extremely uncommon in adults. Central nervous system neoplasms with the combined features of both primitive neuroectodermal tumor and malignant glioma are very rare and represent a diagnostic and treatment predicament. The patient underwent surgical resection, radiation therapy, and chemotherapy targeting both the primitive neuroectodermal tumor and glioblastoma. Our patient has been fortunate in not showing any sign of recurrence and will celebrate the third anniversary since her diagnosis this January.

## Introduction

Collision tumors are characterized by the existence of two distinct neoplasms emerging in the same anatomic location. Such co-occurrence of tumors is rare and even more so in the brain. Our patient was diagnosed with a collision tumor composed mainly of primitive neuroectodermal tumor (PNET) with small areas of glioblastoma (GBM). This brought a poor prognosis and represented a diagnostic and management dilemma. 

## Case presentation

**Figure 1:**
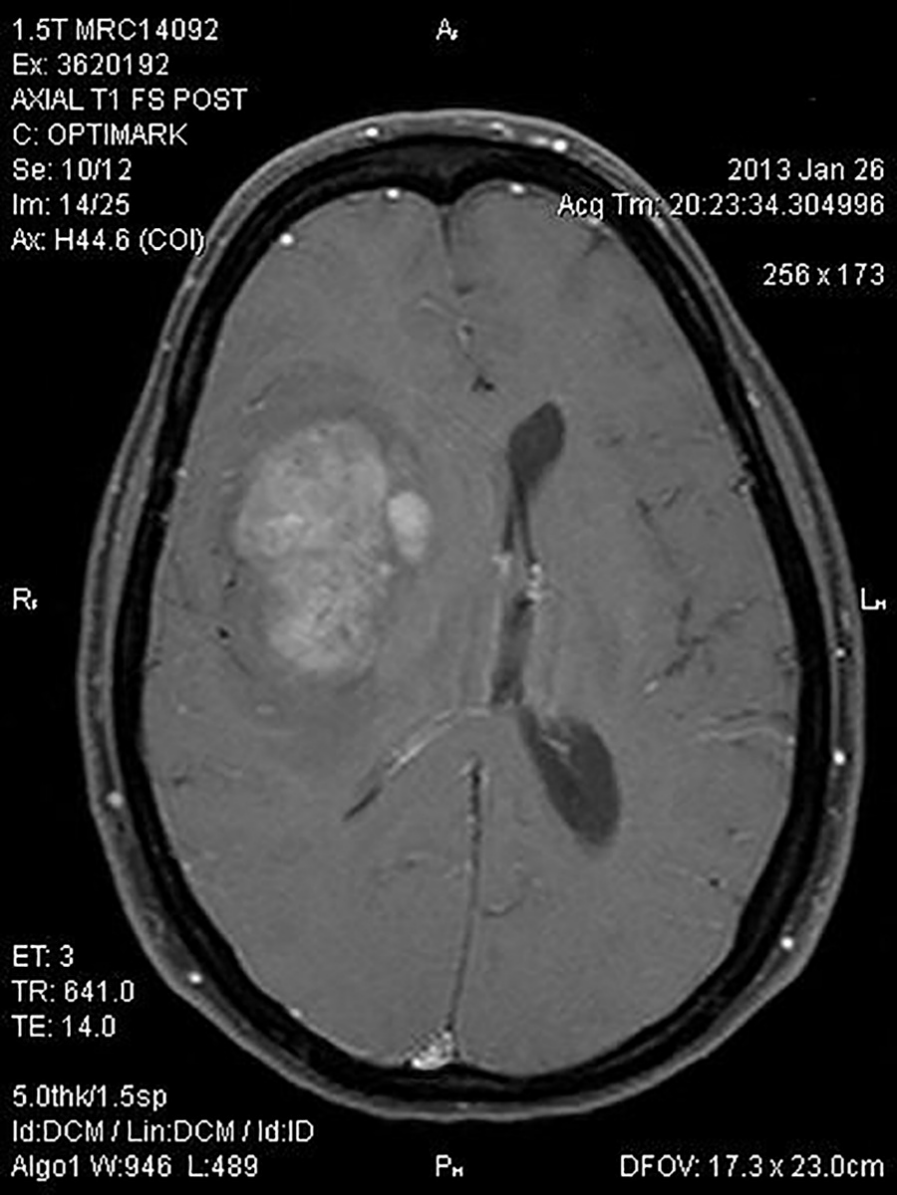
Initial MRI of the brain with and without contrast. A large right frontal lobe intra-axial mass with mass effect upon the right ventricular system and a leftward midline shift of approximately 1.3 cm is shown.

Our patient is a 40-year-old woman who presented with a severe headache and left facial droop in January of 2013. This was accompanied by sensations of strong taste and a pungent odor. She experienced a Jacksonian seizure. A brain MRI revealed a large, right, temporal tumor shown in Figure 1. 

She had a craniotomy with near gross total resection in January 2013. The pathology revealed a primitive neuroectodermal tumor. She experienced left hemiparesis postoperatively. This improved with aggressive rehabilitation, but she remained with left-hand contractures, weakness, and a partial left foot drop.

**Figure 2:**
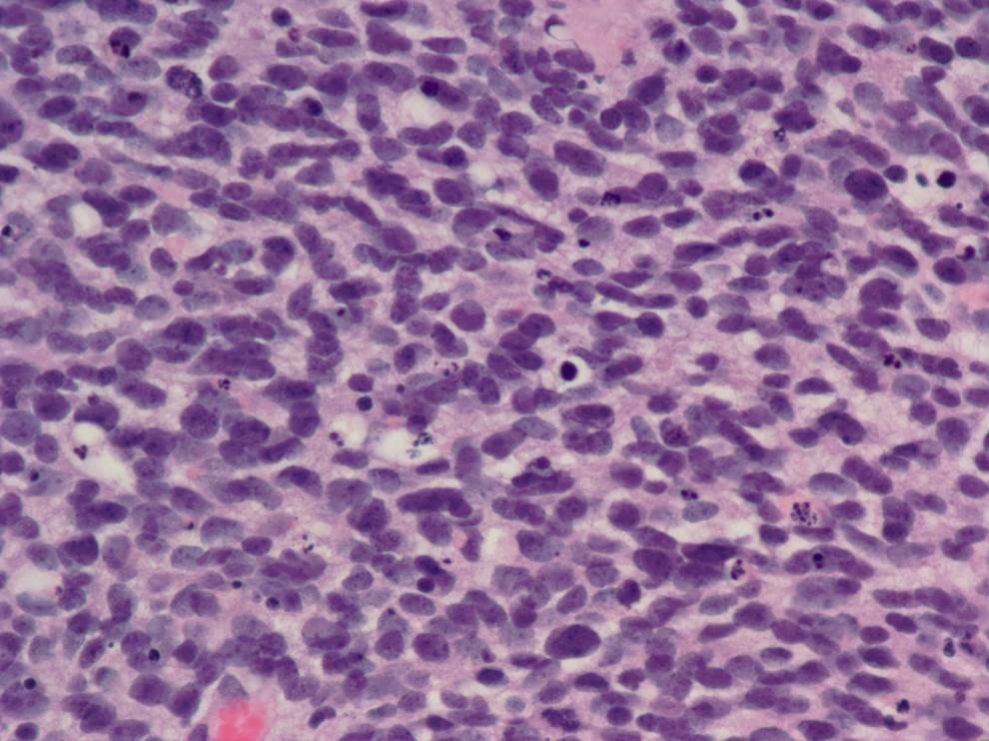
Hematoxylin and eosin staining of the tumor.

**Figure 3:**
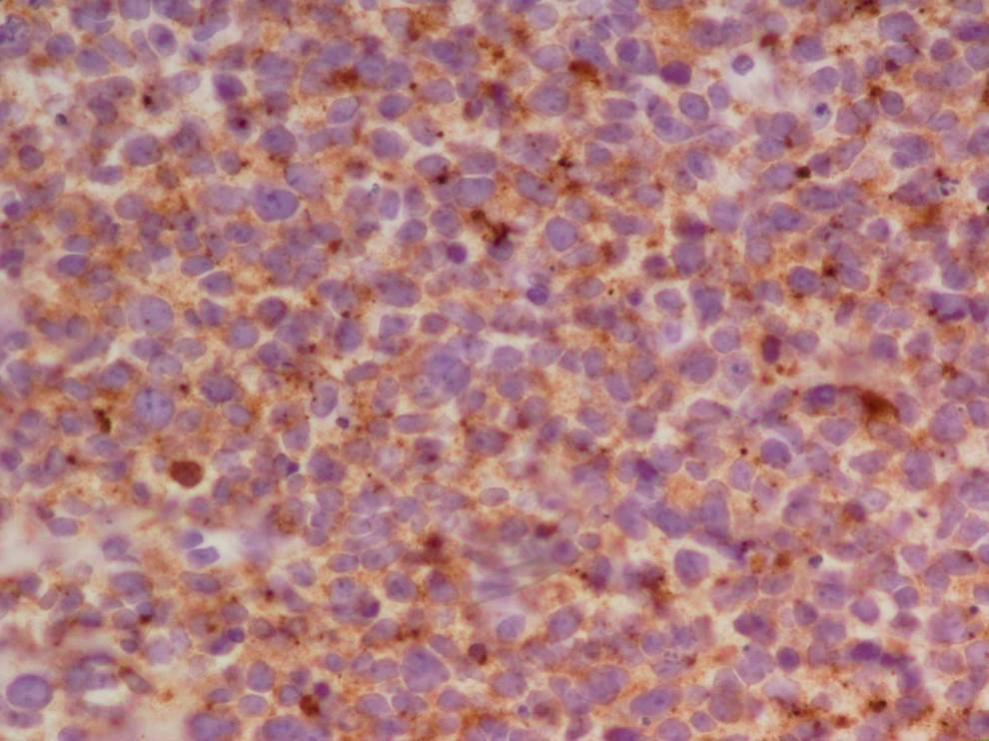
Synaptophysin immunohistochemical staining of the tumor typical of PNET.

She was seen in consultation at The Center for Neuro-Oncology at Dana-Farber. The shared plan was to undergo craniospinal radiation followed by a PNET targeted chemotherapy regimen with cisplatin, vincristine, and cyclophosphamide. Her tumor slides were further reviewed by a neuropathologist at Dana-Farber, and foci of glioblastoma were noted. Her tumor was felt to represent a collision tumor composed of predominately primitive neuroectodermal tumor with small areas of glioblastoma. Selected histological findings are exhibited in Figures 2-3. Not depicted is staining for GFAP, which was positive in approximately 10% of the cells.

She completed her craniospacial radiation therapy, which she tolerated well. She received 12 cycles of alternating chemotherapy. The 1^st^, 4^th^, 7^th^, and 10^th^ cycles were comprised of high-dose cisplatin. She received vincristine only with cycle 1. Due to peripheral neuropathy, she was given high-dose cyclophosphamide during the 2^nd^, 5^th^, 8^th^, and 11^th^ cycles. The 3^rd^, 6^th^, 9^th^, and 12^th^ cycles consisted of oral temozolomide and oral etoposide to target the glioblastoma component of her collision tumor.

**Figure 4:**
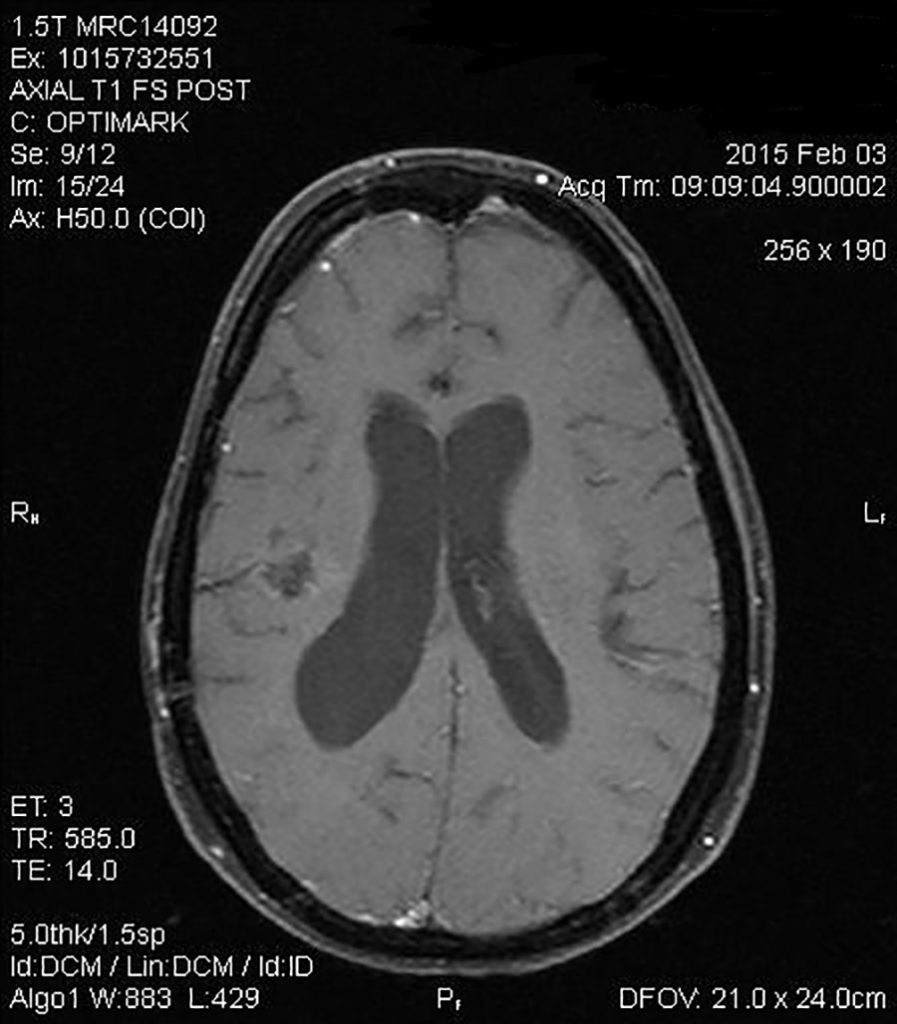
Post-treatment MRI of the brain with and without contrast. Postoperative changes in the right frontotemporoparietal region are shown. Irregular enhancement at the surgical site extends to the walls of the right lateral ventricle. This was unchanged in subsequent MRI scans.

She finished her year of chemotherapy in February of 2014. Her restaging brain MRI shown in Figure 4 was markedly improved without recurrence of the tumor.

Her staging spinal MRI, lumbar puncture, and bone marrow biopsies were all negative. She tolerated the course well overall, but struggled with her left-foot drop and left-hand contracture. She underwent intensive rehabilitation and physical and occupational therapy. She received botox injections at the Yale Movement Disorder Clinic which improved her contractures. Her left-hand function and gait slowly improved with these aforementioned interventions.

She underwent a restaging MRI in June 2015, which demonstrated a stable resection cavity with a decrease in the flair signal. There was no enhancement to suggest a recurrent tumor. She celebrated the anniversaries of her diagnosis and the completion of her treatment. She remains progression-free at this time. She continues to receive regular botox injections. Her gait and function have improved significantly. She continues to perform a vigorous home exercise program and wears an ankle-foot orthosis and wrist splint. Currently, she is able to walk and spend time with her husband and three small children.

## Discussion

Primary tumors of the central nervous system are classified according to the World Health Organization (WHO) guidelines. Glioblastoma multiforme is classified as WHO Grade IV and is one of the most aggressive human malignancies. The highly malignant nature of these tumors makes them difficult to treat. Stupp, et al. revealed that newly diagnosed GBM patients who underwent radiation therapy with concomitant temozolomide had improved median survival (15.6 months versus 12.1 months), two-year survival, three-year survival, and five-year survival versus radiation therapy alone. Radiotherapy along with alkylating chemotherapeutic agents, such as temozolomide, has become the cornerstone of treatment [1-2]. Despite treatment with surgery, radiotherapy, and temozolomide chemotherapy, many of these tumors are resistant to therapy with an average survival of about one year and poor quality of life throughout the course of the disease.

In contrast to GBM, primitive neuroectodermal tumors of the central nervous system are almost exclusively seen in the pediatric population with few cases reported in adults. No optimal treatment regimen has been established. These tumors have a high proliferation rate and often disseminate to the cerebrospinal fluid requiring craniospinal radiation combined with platinum-based chemotherapy. PNETs carry a poor prognosis similar to glioblastoma multiforme, but often have a more robust response to therapy [3]. Kim, et al. reported a 75% three-year mean survival in their series of adult patients [4]. Upon review of several case reports in the literature, most adults with primitive neuroectodermal tumors die within a year of diagnosis.    

Central nervous system neoplasms with the combined features of both malignant glioma and primitive neuroectodermal tumor are rare and are usually reported as single case reports [5-6]. These collision tumors are poorly characterized and are challenging to categorize, posing unique diagnostic and treatment dilemmas. Perry, et al. studied fifty-three cases of brain tumors with the combined features of GBM and CNS PNET. The median survival of these patients was 9.1 months [7].

These CNS collision tumors pose challenges to our patients, the Pathologists who classify them, and the Oncologists who treat them. Despite a poor prognosis and highly aggressive biology, our patient exhibited an exceptional response to therapy that incorporated surgery, radiotherapy, platinum-based chemotherapy to target the propensity for PNET-like CSF dissemination, and temozolomide for glioblastoma multiforme.

## Conclusions

This report illustrates a challenging case in which a patient underwent targeted therapy aimed at treating her primitive neuroectodermal tumor with glioblastoma multiforme components with fortunate results. She had an exceptional response and will celebrate her three year anniversary since her diagnosis in January.
